# Coping profiles and their association with vicarious post‐traumatic growth among nurses during the three waves of the COVID‐19 pandemic

**DOI:** 10.1111/jocn.16988

**Published:** 2024-01-15

**Authors:** Argyroula Kalaitzaki, Maria Theodoratou, George Tsouvelas, Alexandra Tamiolaki, George Konstantakopoulos

**Affiliations:** ^1^ Department of Social Work Hellenic Mediterranean University Heraklion Greece; ^2^ Laboratory of Interdisciplinary Approaches for the Enhancement of Quality of Life, School of Health Sciences Hellenic Mediterranean University Heraklion Greece; ^3^ Institute of AgriFood and Life Sciences Hellenic Mediterranean University Heraklion Greece; ^4^ School of Humanistic Sciences Hellenic Open University Pafos Cyprus; ^5^ School of Health Sciences Neapolis University of Pafos Pafos Cyprus; ^6^ Department of Nursing University of West Attica Thessaloniki Greece; ^7^ Department of Psychiatry National and Kapodistrian University of Athens, Eginition Hospital Athens Greece; ^8^ Department of Clinical, Education and Health Psychology University College London London UK

**Keywords:** cluster analysis, coping strategies, coronavirus, mental health workers

## Abstract

**Aims:**

This study aimed to examine (a) changes in coping strategies and vicarious post‐traumatic growth (VPTG) across three timepoints of the COVID‐19 pandemic among nurses; (b) discrete groups of nurses with unique coping profiles and (c) the association of these coping profiles with VPTG across the timepoints.

**Background:**

Although literature abounds with the negative mental health consequences of the pandemic among healthcare professionals, much less is known about the positive consequences on nurses, the coping strategies that they use, and how these change over time.

**Design:**

This was a cross‐sectional web‐based survey at three timepoints during the pandemic.

**Methods:**

A sample of 429 nurses completed online the Post‐Traumatic Growth Inventory (PTGI) and the Brief Coping Orientation to Problems Experienced Inventory (COPE) to measure vicarious post‐traumatic growth (VPTG) and coping strategies, respectively. The STROBE checklist was used to report the present study.

**Results:**

Significantly higher VPTG scores were observed during the third timepoint. Different coping strategies were employed across the three timepoints. Nurses responded to the pandemic either with an active, an avoidant or a passive coping profile. Significantly higher VPTG levels were reported by the nurses of the active profile compared to those of the passive profile, whereas the difference between active and avoidant profiles was not significant.

**Conclusions:**

Notwithstanding the preponderance of the nurses with the active coping profile in achieving high VPTG, the avoidant copers had more gains (VPTG) than the passive copers, suggesting that doing something to cope with the stressor—let it be trying to avoid it—was better than doing nothing.

**Relevance to Clinical Practice:**

The identification of distinct coping profiles among nurses and their association with VPTG is of particular use to policymakers and practitioners in developing tailored prevention and intervention efforts to help the nurses effectively manage the demands of the pandemic.

**Patient or Public Contribution:**

No patient or public contribution since the study was exclusively conducted by the authors.

**What does this paper contribute to the wider global clinical community?**

- The findings of this study suggest that positive consequences of the pandemic, such as vicarious post‐traumatic growth, are likely among nurses through time.
- Active coping strategies are superior in achieving vicarious post‐traumatic growth than avoiding or non‐responding to the COVID‐19‐related challenges throughout the pandemic.
- Preventive efforts before or immediately at the start of a stressor, such as the pandemic, seem to be more efficient in addressing nurses' coping deficiencies rather than intervention efforts once the stressor has evolved.

## 1 INTRODUCTION

COVID‐19 pandemic has placed immense pressure on healthcare systems worldwide (Kalaitzaki & Rovithis, 2021). Coronavirus has been considered a highly stressful and potentially traumatic experience for all (Bridgland et al., 2021; Kalaitzaki, Tsouvelas, Tamiolaki, & Konstantakopoulos, 2022) and incomparably more challenging and traumatic for healthcare workers (HCWs) (Chutiyami et al., 2022; Kalaitzaki, 2021) and nurses (Kelley et al., 2022). Frontline nurses unevenly suffer the consequences of the pandemic (e.g. stress, depression, anxiety, burnout; Fernandez et al., 2021; Guastello et al., 2022; Mosolova et al., 2021; Sagherian et al., 2022) and are more seriously affected than the rest of the nurses and HCWs in general (Chutiyami et al., 2022; De Kock et al., 2021; Perego et al., 2022). Studies have also shown that nurses are at high risk of secondary traumatic stress (İlhan & Küpeli, 2022).

In the face of highly stressful and/or traumatic experiences, positive impacts on people's mental health have also been reported. Vicarious post‐traumatic growth (VPTG) has been defined as the positive changes that HCWs may indirectly gain from working directly with victims of trauma (Chen et al., 2021), such as increased appreciation of life, improved relationships with others, enhanced spiritual faith, empowerment of the self and discovery of new possibilities (Tedeschi & Calhoun, 2004). It has been associated with less depression and anxiety, increased well‐being and life satisfaction (Helgeson et al., 2006) and better psychological adjustment (Aggar et al., 2022) in nurses. At the beginning of the pandemic, low‐to‐moderate levels of post‐traumatic growth were reported in HCWs (Kalaitzaki et al., 2020; Kalaitzaki, Tamiolaki, & Tsouvelas, 2022) and in specific professional groups such as nurses (Cui et al., 2021; Li et al., 2022; Mo et al., 2022; Yeung et al., 2022; Zhang et al., 2021). As VPTG is a dynamic process and prevalence rates may vary considerably across different timepoints (Manning‐Jones et al., 2016), examining PTG immediately after a traumatic event is one of the main criticisms. Only a few studies so far have examined VPTG among HCWs. Kalaitzaki et al. (2023) found higher VPTG levels during the second timepoint, with medical HCWs (i.e. physicians and nurses) reporting increased VTPG and non‐medical HCWs (i.e. psychologists and social workers) reporting decreased VTPG at the second timepoint. Other studies have found decreased PTG among HCWs over two (Yılmaz‐Karaman et al., 2022) or three timepoints (Lyu et al., 2021) or various trajectories, ranging from low to high, with increases or fluctuations (Yan et al., 2022). More research in this field is necessary.

How do HCWs cope with the pandemic to obtain positive outcomes, such as VPTG? Among various protective factors (e.g. resilience, coping strategies and social support), coping strategies are the most studied personal resources (Finstad et al., 2021). Coping has been defined as “constantly changing cognitive and behavioural efforts to manage specific external and/or internal demands that are appraised as taxing or exceeding the resources of the person” (Lazarus & Folkman, 1984, p. 141). A growing body of literature has examined the association of coping strategies with purely positive mental health outcomes in nurses, such as VPTG (Li et al., 2022); but the findings are still inconclusive. In HCWs generally, a variety of coping strategies, either problem‐focused or emotion‐focused (Hamama‐Raz & Minerbi, 2019; Ogińska‐Bulik & Zadworna‐Cieślak, 2018), adaptive or maladaptive (Asmundson et al., 2021; Kalaitzaki & Rovithis, 2021; Kalaitzaki, Tamiolaki, & Tsouvelas, 2022) have been associated with PTG during the pandemic. Moreover, coping strategies have been found to mediate the relationship between HCWs' mental health and PTG (Prekazi et al., 2021), and adaptive coping strategies to mediate the relationship between secondary traumatic stress and VPTG (Kalaitzaki et al., 2023; Kalaitzaki, Tamiolaki, & Tsouvelas, 2022), suggesting that growth rather than resulting directly from trauma occurs as a result of people's coping efforts with the trauma.

Although there are many longitudinal studies examining coping strategies, they have mostly examined their role in the well‐being and psychological health of HCWs (Chew et al., 2020; Elliott et al., 2021; Foster et al., 2022; Perego et al., 2022; Rodríguez‐Rey et al., 2022; Zhou et al., 2021), and not whether they change over time. Some studies have examined coping strategies over different periods of the pandemic but in populations other than HCWs (e.g. general population, Kalaitzaki, Tsouvelas, & Tamiolaki, 2022; older people, Kuo et al., 2021; college students, Rogowska et al., 2021). By the writing of this paper, only one study has examined changes in coping strategies between waves or lockdowns among HCWs (Manara et al., 2021) and another one among nurses (Van Steenkiste et al., 2022). Minor changes in the approach coping strategy over a week of daily assessments were found among HCWs (Manara et al., 2021) with slightly higher scores among the younger HCWs (aged equal or under 38 years), and no significant changes in the avoidance coping strategy over three timepoints (recruitment, 4 and 8 weeks later) were found among nurses in the longitudinal study by Van Steenkiste et al. (2022).

Studies have also shown that the efficiency of the coping strategies depends at least partially on the specific stressor (Kavčič et al., 2022); e.g. providing temporary relief, maladaptive coping strategies could be effective in dealing with uncontrollable stressors, such as infectious diseases (Kalaitzaki et al., 2023; Kalaitzaki, Tsouvelas, & Tamiolaki, 2022). Whereas the use of adaptive coping strategies by HCWs has been associated mostly with better psychological functioning and the use of maladaptive coping strategies with worse psychological functioning in the long run (Mong & Noguchi, 2021), it has also been shown that a seemingly maladaptive strategy could likely have short‐term benefits and adherence to a seemingly adaptive strategy could likely have long‐term negative outcomes, such as deteriorating mental health and negative adjustment (Kalaitzaki et al., 2023; Ziarko et al., 2022). Since time needs to be considered when examining the effectiveness of the coping strategies, the association of the coping strategies with VPTG among nurses at different time points during the current pandemic is an important aim of this study. Furthermore, Pearlin and Schooler (1978) have suggested that a single coping response towards life strains, regardless of its own efficacy, might be less effective than a variety of strategies. Thus, it could be argued that a variety of weapons might be more beneficial than having a particular weapon in one's supplies. It is, therefore, necessary to examine specific coping profiles (complexes of strategies) among nurses during COVID‐19, their association with VPTG, and potential longitudinal changes.

This study aimed to (a) explore frequently used coping strategies and VPTG rates among nurses across the three waves, (b) determine whether nurses could be differentiated based on unique combinations of coping strategies (coping profiles) and (c) examine the relationships between these coping profiles and VPTG. Based on the literature (Kalaitzaki et al., 2023; Mo et al., 2022) we hypothesised that (a) increased VPTG and more adaptive than maladaptive coping strategies are expected across time; (b) nurses will present distinct coping profiles and (c) these coping profiles will be differentially associated with VPTG fluctuations. The identification of distinct coping profiles among nurses who share similar coping characteristics would provide insights into the complexity of coping and would be of particular use for policymakers and practitioners who develop interventions to promote VTPG in unique groups of nurses.

## 2 METHOD

This study used the STrengthening the Reporting of Observational studies in Epidemiology (STROBE) guidelines in reporting methods and findings (Vandenbroucke et al., 2007) (Appendix S1).

### 2.1 Participants

From the vast number of nurses approached (see Study design and procedure), eventually a total of 429 nurses were recruited from different regions of Greece at three timepoints (*Ν* = 222 in April 2020, *Ν* = 108 in November–December 2020, and *Ν* = 99 in May 2021). The inclusion criteria were: nurses residing in Greece, working in any healthcare facility (e.g. hospital, community center), who comprehended Greek and gave informed consent. Nurses who were not currently working were excluded. After controlling for outliers with anomaly detection techniques, no cases were excluded. They were mostly females (87.4%), married (62.7%), with a mean age of 41.8 years (SD = 9.2; min = 22, max = 65) and diverse working experience (53.8% up to 15 years). The detailed sociodemographic characteristics of the overall sample can be seen in Table 1.

**TABLE 1:** Sociodemographic characteristics of the sample.

	Total (*n* = 429)		T1 (*n* = 222)		T2 (*n* = 108)		T3 (*n* = 99)	
	*f*	%	*f*	%	*f*	%	*f*	%
Sample	429	100.0	222	51.7	108	25.2	99	23.1
Gender								
Male	54	12.6	28	12.6	15	14.0	11	11.1
Female	374	87.4	194	87.4	92	86.0	88	88.9
Age	41.8	9.2	42.3	8.9	40.1	9.4	42.4	9.5
Marital status								
Single	115	26.8	54	24.3	38	35.2	23	23.2
Married	269	62.7	137	61.7	60	55.6	72	72.7
Other (divorced, widow)	45	10.5	31	14.0	10	9.3	4	4.0
Contact with confirmed patients								
Νο	103	24.0	70	31.5	24	22.2	9	9.1
Yes	326	76.0	152	68.5	84	77.8	90	90.9
Educational level								
Technical education	91	21.2	53	23.9	8	7.4	30	30.3
University education	119	27.7	114	51.4	55	50.9	50	50.5
Master/Doctoral	94	21.9	55	24.8	45	41.7	19	19.2
Work experience								
Up to 5 years	94	21.9	38	17.1	30	27.8	26	26.3
6 to 10 years	43	10.0	27	12.2	11	10.2	5	5.1
11 to 15 years	94	21.9	54	24.3	18	16.7	22	22.2
16 to 20 years	67	15.6	40	18.0	12	11.1	15	15.2
21 to 25 years	57	13.3	31	14.0	15	13.9	11	11.1
26 to 30 years	21	4.9			9	8.3	12	12.1
More than 31 years	53	12.4	32	14.4	13	12.0	8	8.1

*Note*: Age is presented in mean and standard deviation.

### 2.2 Study design and procedure

**FIGURE 1:**
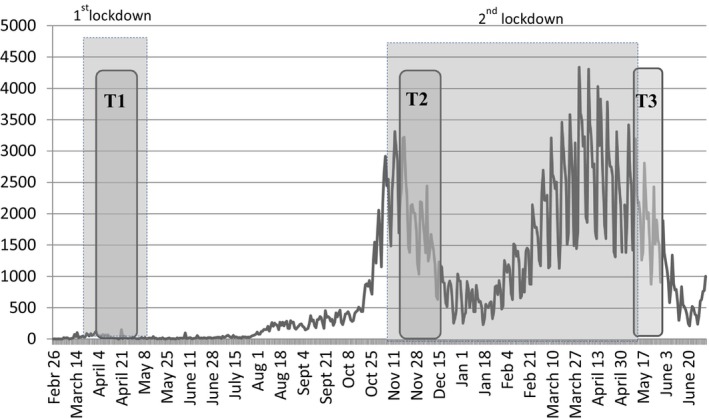
The three timepoints of recruitment during the course of the daily numbers of confirmed COVID‐19 cases in Greece. Data from https://covid19.gov.gr/covid19‐live‐analytics/. [Colour figure can be viewed at wileyonlinelibrary.com]

This was a nation‐wide cross‐sectional study repeated at three timepoints (see Figure 1): April 2020 (T1), 15 November–15 December 2020 (T2) and 15–30 May 2021 (T3). T1 (April 2020) was in the middle of the first lockdown in Greece (23 March to 03 May 2020), which was proactively imposed by the government, T2 (15 November–15 December 2020) was at the beginning of the second lockdown (7 November 2020 to 15 May 2021) and T3 (15–30 May 2021) after the end of the second lockdown.

The convenience and snowball mixed sampling procedure were the methods of recruitment. Anonymized questionnaires were administered both in person and online by the authors and their colleagues. The online survey with the study information and the questionnaire link via shortened Uniform Resource Locators (URLs) were circulated by the authors and their colleagues through emails, social media accounts and platforms (Facebook and Instagram), Greek news portals and professional webpages. The potential participants who were approached either online or in person, were kindly asked to fill in the questionnaire and share the link of the survey with their acquaintances, colleagues and social media contacts, who were nurses residing and working in any place in Greece. On the first page of both the online and hardcopy questionnaire, participants were presented with a complete description of the survey and informed consent with their rights (voluntary and anonymous participation, right to withdraw). Informed consent was obligatory for one to continue completing the survey. The study abided with the 1964 Helsinki Declaration and its later amendments, and all procedures were approved by the Research Ethics Committee of the Hellenic Mediterranean University.

### 2.3 Measures

The questionnaire asked demographic questions such as age, gender, marital status, education, work experience and a number of questions about nurses' experience with COVID‐19 (i.e. whether they had contact with confirmed COVID‐19 cases, level of self‐efficacy of implementing the COVID‐19 protocols).

The Post‐Traumatic Growth Inventory (PTGI) (Tedeschi & Calhoun, 1996), consisting of 21 items and allocated in five domains (personal strength, relating to others, new possibilities, appreciation of life and spiritual change), measured contingent growth that occurred following the COVID‐19 pandemic. Sample items include ‘I have a greater appreciation for the value of my own life’ (appreciation of life) and ‘I more clearly see that I can count on people in times of trouble’ (relating to others). Items were scored on a 6‐point scale, ranging from 0 (I did not experience this change) to 5 (I experienced this change to a very large extent). A total score ranging from 0 to 105, and five subscale scores were produced, with higher scores indicating higher levels of growth. Applying the cut‐off score criterion of 46, two levels of PTG can be identified; scores ≤45 indicate none/low PTF whereas scores ≥46 indicate medium/high PTG (Mazor et al., 2016). The reliability (Cronbach's *α*) of the PTGI in the current sample was .95.

The Brief COPE (Coping Orientation to Problems Experienced Inventory; Carver et al., 1989), consisting of 28 items and sorted into 14 subscales of two items each (i.e. Active coping, Planning, Emotional support, Instrumental support, Positive reframing, Acceptance, Religion, Humour, Venting, Denial, Substance use, Behavioural disengagement, Self‐distraction, Self‐blame), was used to determine the frequency of effective/adaptive (the first eight) and ineffective/maladaptive ways (the remaining) to cope with the COVID‐19 pandemic. Sample items include ‘I've been taking action to try to make the situation better’ (problem‐focused coping), ‘I've been getting emotional support from others’ (emotion‐focused coping) and “I've been giving up trying to deal with it” (dysfunctional coping). The items were scored on a four‐point scale ranging from 1 (I haven't been doing this at all) to 4 (I've been doing this a lot) and 14 subscale scores were produced. The reliability (Cronbach's *α*) of the Brief COPE was .84.

### 2.4 Statistical analyses

Independent samples *t*‐tests were performed to test gender differences on VPTG and coping strategies. A series of one‐way Analyses of Covariance were conducted to test differences in coping strategies and VPTG between the three timepoints, while controlling for relationship status (married/in a relationship vs single/divorced/other) as a covariate. Scores above threshold 46 were used to determine moderate to very high VPTG levels (Mazor et al., 2016). A hierarchical cluster analysis was conducted using a two‐step process to identify distinct coping profiles between nurses. After identifying the coping profiles, a two‐way Analysis of Covariance examined the effect of timepoint and clusters of coping strategies on VPTG with the relationship status (married/in a relationship vs single/divorced/other) as a covariate. IBM SPSS v23 was used and all analyses with a *p*‐value < .05 were considered statistically significant. Partial eta squared (*η* ^2^) and Cohen's d provided indices of effect size.

## 3 RESULTS

### 3.1 VPTG, use of coping strategies and differences across the three timepoints

ANCOVA showed differences in VPTG mean scores across timepoints with higher overall and subscales scores during T3 (except for ‘Spiritual Change’ and ‘Appreciation of Life’) compared to T1 and T2 after controlling for relationship status (see Table 2). Taking into consideration the cut‐off criterion 46, there were statistically significant VPTG differences across timepoints (*χ* ^2^_(2)_ = 8.97, *p* = .011), with more nurses presenting medium/high VPTG scores at T3 (76.8%) than T1 (60.4%) and T2 (60.2%). It was also shown that emotional support, instrumental support and self‐blame were more frequently used at T2 and T3 in comparison with T1, substance use, and planning were more frequently used at T2 in comparison with T1 and T3, and venting was more frequently used at T3 compared to T1 after controlling for relationship status (Table 2).

**TABLE 2:** Differences in coping strategies and VPTG across the three timepoints after controlling for relationship status.

	Adjusted means					
	T1	T2	T3	*F*	*p*	*η* ^2^
COPE self‐distraction	6.26	5.98	6.17	1.64	.195	.008
COPE active coping	5.82	6.11	6.09	2.19	.113	.010
COPE denial	3.95	4.00	3.81	.38	.69	.002
COPE substance use	2.29_b_	2.60_a_	2.23_b_	4.26	.015	.020
COPE use emotional support	4.82_b_	5.35_a_	5.66_a_	9.30	.000	.042
COPE use instrumental support	4.79_b_	5.31_a_	5.76_a_	11.51	>.001	.051
COPE behavioural disengagement	2.69	2.97	2.93	2.526	.081	.012
COPE venting	5.05_b_	5.44_ab_	5.61_a_	5.08	.007	.023
COPE positive reframing	6.21	6.22	6.05	.49	.610	.002
COPE planning	6.13_ab_	6.40_a_	5.71_b_	5.63	.004	.026
COPE humour	4.38	4.62	4.74	1.99	.138	.009
COPE acceptance	6.26	5.98	6.17	1.64	.195	.008
COPE religion	4.52	4.43	4.59	.18	.837	.001
COPE self‐blame	3.86_b_	5.11_a_	4.63_a_	27.87	>.001	.116
VPTG relating to others	15.40_b_	16.81_b_	20.84_a_	12.89	>.001	.057
VPTG new possibilities	10.86_b_	11.95_ab_	13.86_a_	7.76	>.001	.035
VPTG personal strength	11.53_b_	11.75_b_	13.39_a_	4.89	.008	.022
VPTG spiritual change	4.55	5.07	4.85	1.08	.339	.005
VPTG appreciation of life	9.00	9.01	9.73	1.27	.281	.006
VPTG total	51.33_b_	54.59_b_	62.66_a_	7.63	.001	.035

*Note*: Adjusted means with different scripts differ at the *p* = .05 according to Bonferroni. Relationship status (0 = single/without relationship, 1 = married/in a relationship) was the covariate.

### 3.2 Cluster analysis on coping strategies

**FIGURE 2:**
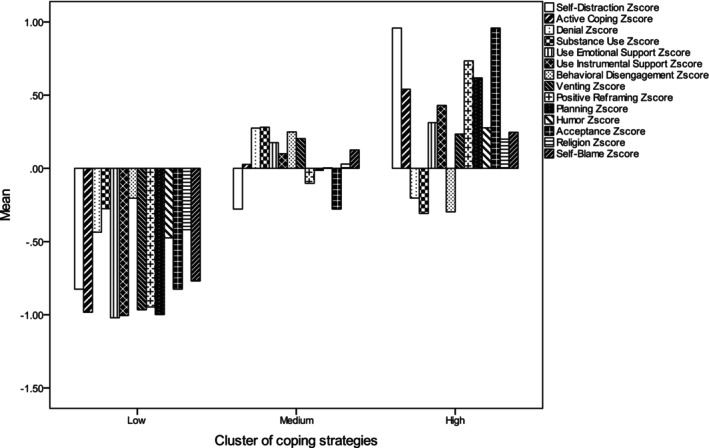
Distribution of the clusters of coping strategies.

A cluster analysis (using Ward's method) was conducted to identify groups of nurses in terms of the coping strategies they use. Α hierarchical cluster analysis using Ward's criterion was initially used to identify the pairs of clusters that result in a minimum increase of the total within‐cluster variance after merging (Borgen & Barnett, 1987; Ketchen Jr. & Shook, 1996). From the solutions of two or three cluster profiles, we chose the 3‐clusters as best fitting the aims of the study (i.e. being detailed enough and yet providing a robust number of cases in each cluster for further analysis). Three clusters of coping strategies were extracted (see Figure 2): (a) low coping representing 78 nurses (18.2%) who had low scores in all coping strategies, (b) moderate coping representing 220 nurses (51.3%) who had moderate scores in most of the coping strategies (e.g. substance use, denial, emotional and instrumntal support and self‐blame) and low scores in self‐distraction, positive reframing, planning and acceptance and (c) high coping representing 131 nurses (30.5%) who had high scores in nearly all coping strategies and low scores in denial, substance use and behavioural disengagement.

### 3.3 Differences in VPTG by timepoint and clusters of coping strategies

**FIGURE 3:**
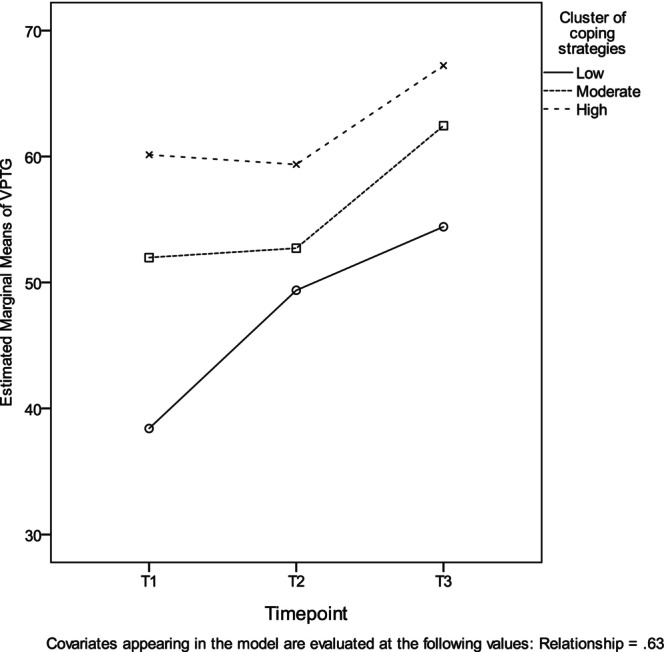
Interaction effect of timepoints (T1, T2, T3) and clusters of coping strategies (low, moderate, high) on VPTG, after controlling for relationship status.

A two‐way Analysis of Covariance 3 × 3 examined the effect of Timepoint (T1, T2 and T3) and clusters of coping strategies (low, moderate, high) on VPTG, after controlling for relationship status. There was a significant main effect of timepoint (*F*_(2,419)_ = 5.96, *p* = .003, *η* ^2^ = .03) and clusters of coping (*F*_(2,419)_ = 7.69, *p* < .001, *η* ^2^ = .04) on VPTG, but the interaction between the effects of the two independent variables on VPTG was not statistically sign*pM*ificant (*F*_(4,419)_ = .65, *p* = .630, *η* ^2^ = .01). According to Bonferroni post hoc criterion, at T3 nurses reported higher VPTG scores (*M* = 61.36, SE = 2.81) compared to T1 (*M* = 50.17, SE = 1.63), and nurses in the high coping cluster reported higher VPTG scores (*M* = 62.24, SE = 2.18) compared to nurses in the low coping cluster (*M* = 47.40, SE = 3.18). VPTG scores at T2 ( = 53.82, SE = 2.65) did not differ from T1 and T3 and VPTG scores in the moderate cluster (*M* = 55.71, SE = 1.63) did not differ from the low and high clusters. Figure 3 presents the effects of timepoints and clusters of coping strategies on VPTG.

## 4 DISCUSSION

The aims of this study were to examine rates of VPTG, and frequency of coping strategies used among nurses across three timepoints of the pandemic, identify specific coping profiles (combinations of strategies) and their association with VPTG. Significantly higher VPTG scores were observed during the third timepoint of the pandemic and different coping strategies were employed across the three timepoints. Nurses adopted either with an active, an avoidant or a passive coping profile, which correlated with high, moderate or low VPTG levels, respectively.

Consistent with the expectations (Kalaitzaki et al., 2023; Manning‐Jones et al., 2016) nurses reported higher VPTG scores at T3 compared to T1. It seems that as time passes by, VPTG increases progressively, which could be an indication of real and not illusory PTG (Lyu et al., 2021; Zoellner & Maercker, 2006). Kalaitzaki et al. (2023) have also found higher VPTG in T2, compared to T1, among HCWs. Since Yan et al. (2022) found that individual factors, such as age, education and resilience correlate with different PTG trajectories (i.e. persistent low, steady increase, high with drop, fluctuated rise) in frontline HCWs, future studies should take into consideration the duration of traumatic event along with sociodemographic characteristics.

Similarly, the frequency of using specific coping strategies changed across the three timepoints and thus, different coping strategies were used at different timepoints. Although other studies have not found significant differences over time, it may be so because they studied only the predominant coping strategy of avoidance (Van Steenkiste et al., 2022) or the timepoints were too short (Manara et al., 2021). In this study, emotional and instrumental support and self‐blame were more frequently used at later timepoints (T2 and T3), substance use and planning were more frequently used at T2, and venting was more frequently used at T3. This is in keeping with the literature (Kalaitzaki et al., 2023; Vagni et al., 2020) showing that the use of coping strategies depends on the specific features of the pandemic, the perceived control over the situation, the perceived threat and a number of other personal and situational factors. It is quite plausible that when personal resources are considered insufficient in the later stages of the pandemic, HCWs seek for external resources, such as support (Chutiyami et al., 2022; Sehularo et al., 2021). Emotional support may be useful when needing to urgently cope with an unexpected stressor, whereas instrumental support may be useful when people are overwhelmed and potentially have to deal with various deficits (Kalaitzaki et al., 2023). In line with other studies, increased use of substances (Asmundson et al., 2021; Searby et al., 2022) and planning (Salman et al., 2022) were observed in the middle stages of the pandemic. It seems that nurses seek for temporary relief from the stressor, while at the same time they do not abdicate but keep on planning how to deal with the demands. Self‐blame may represent internal locus of control, or else, one's responsibility to cope with stressors and/or disappointment when efforts are not effective (e.g. abide with precautionary measures, remain COVID‐free and in a good psychological state); reasonably enough, self‐blame was more frequently used at later timepoints. Venting may well represent nurses' exhaustion and overburden and the necessity to release overwhelming emotions. Nurses' high VPTG scores suggest that the combinations of the so‐called adaptive and maladaptive coping strategies are likely to be effective at different timepoints of the pandemic. Thus, the distinction between adaptive versus maladaptive coping may not be accurate and useful. Effective versus ineffective coping strategies could be a meaningful variant.

A notable finding of this study was the identification of three distinct clusters of coping strategies: i.e. three coping profiles that consist of different combinations of so‐called adaptive and maladaptive strategies. Somewhat surprisingly, a small proportion of the nurses (18.2%) made nearly no or extremely infrequent use of any coping strategy. This may represent what Kavčič et al. (2022) have called the disengaged profile of people who make low use of all strategies. A high proportion of the nurses (51.3%) had moderate scores in most of the coping strategies, predominantly maladaptive (i.e. substance use, denial, behavioural disengagement, venting, self‐blame and emotional and instrumental support) and low scores in predominantly adaptive coping strategies (i.e. positive reframing, planning, acceptance and self‐distraction). We call them ‘avoidant copers’, following Kavčič et al. (2022) classification. Νearly one‐third of the nurses, the ‘high copers’, were frequently employing a compilation of primarily so‐called adaptive coping strategies (i.e. acceptance, positive reframing, planning, active coping, instrumental and emotional support, humour, religion also self‐blame, self‐distraction and venting) and rarely the so‐called maladaptive coping strategies (i.e. denial, substance use and behavioural disengagement). People with the combination of these predominantly adaptive coping strategies have been called engaged copers (Kavčič et al., 2022). We would rather suggest the term ‘active copers’; they accept what they cannot control or change, and they are actively involved in finding ways by all means to overcome stress and effectively adjust to the new challenges.

In line with findings suggesting that the so‐called adaptive coping is associated with favourable mental health outcomes whereas the so‐called maladaptive coping is associated with unfavourable mental health outcomes (Alaradi et al., 2021; Sehularo et al., 2021), this study found that the nurses with the ‘passive’ coping profile had the lowest levels of VPTG, the nurses with the ‘avoidant’ coping profile had moderate levels of VPTG, and the nurses with the ‘active’ coping profile had the highest levels of VPTG across all three timepoints; the difference was significant between the ‘passive’ and ‘active’ coping profile. Kavčič et al. (2022) have shown that the engaged profile was related with the highest levels of well‐being (positive emotions, engagement, relationships, meaning and accomplishment) and the lowest levels of ill‐being (depression, anxiety and stress), whereas the disengaged and avoidant profiles had the lowest levels of well‐being and the highest levels of anxiety and stress, respectively. The association of the coping profiles with VPTG ascertains our suggestion that coping strategies should rather be called effective/ineffective and is another indication that the VPTG obtained by HCWs was real and not illusory (Kalaitzaki, Tsouvelas, & Tamiolaki, 2022; Zoellner & Maercker, 2006).

Interestingly, the nurses with the ‘active’ coping profile (high coping cluster) had higher VPTG scores at T1, followed by the nurses with the ‘avoidant’ and the ‘passive’ coping profiles (moderate and low coping clusters, respectively), and this pattern continued across the following two timepoints (T2 and T3). Whereas the high and moderate clusters had approximately stable VPTG scores at T2, they raised at T3 to overcome scores at T1. Although it could be assumed that nurses with high coping skills (active coping profile) had better psychological functioning, such as VPTG, from the beginning of the COVID‐19 pandemic and were more qualified to achieve further positive outcomes, the vice versa could be also true; nurses with high VPTG may have had more resources (i.e. VPTG) to better cope with the pandemic.

The low coping cluster (passive) related to the most reduced VPTG compared to the high coping cluster (active) across the three timepoints, and although it did not significantly differ from the moderate cluster (avoidant), doing nothing to cope with the stressor was worse than doing something: the avoidant group of nurses seem to employ a constellation of mostly ineffective strategies, which was more effective (though not statistically significant) for boosting their VPTG than the passively coping group. Therefore, employing mostly ineffective coping strategies (avoidant profile), though not significantly different, seemed better than passively cope (i.e. the avoidant copers had higher VPTG scores across all timepoints than the passive copers). Interestingly, the passive copers had a significant increase in VPTG scores across time, which, however, did not reach that of the other two groups. It can be suggested that having minimum resources (coping skills) from the start cannot compensate for the VPTG gains achieved through time by those already having the appropriate coping skills.

This study has a number of limitations. Despite the repeated cross‐sectional survey methodology, we do not know exactly how many participants were the same between the three timepoints. Future longitudinal studies should examine the evolution of the same participants across three consecutive timepoints. Neither causal inferences nor generalizability of the findings are permitted with such a methodology. The use of a convenience sample may have decreased the representativeness of the sample (e.g. more novice nurses with high educational level). Notwithstanding that the nurses in Greece are still predominantly females, overwhelmingly more female than male nurses were recruited. The two different modes of questionnaire administration (online and offline), and the self‐report format of the questionnaire may have resulted in associated bias (e.g. selection bias and social desirability). We do not know whether participants had in advance coping skills to deal with the stressors or whether they owned other personal resources (e.g. resilience) that may have acted additively to VPTG. More timepoints would be needed to examine the process of VPTG and coping strategies. A larger sample would allow for more comparisons in terms of the demographic data. Further longitudinal studies in large samples examining additional variables are required.

## 5 RELEVANCE TO CLINICAL PRACTICE

The nurses constitute the majority of the healthcare workforce and their role in the healthcare system is critical, but their needs during COVID‐19 have been largely overlooked (Maben & Bridges, 2020). The findings of the present study suggest that they need to be included in any planning or decision‐making. The findings of this study suggest that positive impacts of the pandemic, such as vicarious post‐traumatic growth, are likely among nurses over time. The findings further contribute to the scientific knowledge of how specific constellations of coping strategies are employed by nurses in response to large scale stressors, such as the COVID‐19 pandemic. The less efficient ones (passive and avoidant copers) may require timely interventions that promote active coping with stressors and help them to achieve VPTG. Importantly, the findings of this study suggest that preventive efforts before or immediately at the start of a stressor could likely be more efficient in addressing nurses' coping deficiencies rather than intervention efforts once the stressor has evolved. Nurses are among those professional groups that are highly exposed to COVID‐19‐related hazards and both healthcare organisations and policy efforts should provide them with the support that enables them to perceive moments of extreme crisis as opportunities to grow.

## AUTHOR CONTRIBUTIONS

All authors listed have made substantial contributions to the manuscript to meet the authorship criteria according to the latest guidelines of the International Committee of Medical Journal Editors. AK contributed to the conception and design of the study. AK supervised data collection. GT organised the database and performed the statistical analyses. AK, GT, GK interpreted the results. AK, MT, AT, GT and GK contributed to the first drafting of the manuscript. GK critically reviewed the manuscript. AK and GK edited the final revised version. All authors have read, reviewed and agreed to the final version.

## CONFLICT OF INTEREST STATEMENT

The authors declare that they have no conflict of interest to declare.

## FUNDING INFORMATION

This study received no funding.

## Supporting information


Appendix S1.

## Data Availability

The data supporting the findings of this study are not publicly available due to privacy or ethical restrictions. However, the data will be made available by the corresponding author upon reasonable request and without undue reservation.
